# Extract of Beefs’ Blood in the Treatment of Anæmia

**Published:** 1851-11

**Authors:** 

**Affiliations:** Vienna


ARTICLE V

*Extract of Beefs' Blood in the treatment of Anæmia.* By Dr. Von Mauthner, of Vienna. Translated from the French by H. A. Johnson, A. B., Interne to Illinois General Hospital.

Dr. Von Mauthner, Director of the Hospital, St. Amne, of Vienna, has employed for some time the extract of Beef's Blood in the protracted Anæmias of children. According to this distinguished practitioner a large number of diseases are caused by an Anæmic state, rather than is generally believed, by irritation, and ought, therefore to be treated by other than antiphlogistic means. Unfortunately, science has furnished, as yet but few remedies, capable of combatting successfully Asthenic diseases, having their point of departure in the constitution of the blood.

M. Von M. has employed with success the Ammonio Chloride of Iron in the treatment of children, presenting periodical symptoms of congestion, without any appreciable organic cause, and in debility attending intermittants, but he has become convinced that there are Anæmic conditions in which the patients do not bear the use of any of the preparations of Iron, and it is in such states that the Extract renders the most efficient service.

The extract is prepared in the following manner: Blood, fresh from the animal is thrown in a filter, and the residue evaporated to complete dryness. It is administered in the form of powder, or dissolved in water, in quantities of from grs. 10 to ʒi. per day. Under the continued use of this means the patients improve very much in appearance and gain rapidly in strength. This result ought to astonish no one when it is considered that the extract supplies just those substances which are wanting in the blood of these little sufferers, viz: the hæmatine and the fibrine.

According to Dr. Von Mauthner, this preperation is especially adapted to the following Anæmic morbid conditions:—1st. Anæmia succeeding chronic diarrhœaofchildren of a certain age. It is on the contrary of but very little use in very young subject and in such as have just been taken from the breast. 2d. Anæmia after Typhus. The author who is perfectly convinced of the advantages which it offers in this case, assures us that it may be administered without any danger of fatiguing the digestive organs. 3d. Anæmia which follows severe pneumonia, when the lungs are not as yet restored to their normal state, and the patient is troubled with cough and fever; but it is to be remarked that the remedy is not equally beneficial in tuberculosis. 4th. Anæmia succeeding wasting supperation, and scrofulous ulcers. 5th. Anæmia after serous accumulations produced by scarlatina. In this condition of the system it seems to surpass all other remedies in use, since, contrary to the effect which has been observed of other tonics, it produces no irritation of the kidneys, a result often leading to hæmaturia and albumenuria and constituting a new disease.

This remedy, so simple in its use, and costing only the labor of preparing it, merits, as it seems to us, the attention of practitioners, especially for the poorer classes, among whom Anæmic effectations are unfortunately so common.

*Anales, Med. de la Flandre Ocid.*

